# Acrolein-Induced Oxidative Stress and Cell Death Exhibiting Features of Apoptosis in the Yeast *Saccharomyces cerevisiae* Deficient in SOD1

**DOI:** 10.1007/s12013-014-0376-8

**Published:** 2014-11-14

**Authors:** Magdalena Kwolek-Mirek, Renata Zadrąg-Tęcza, Sabina Bednarska, Grzegorz Bartosz

**Affiliations:** Department of Biochemistry and Cell Biology, University of Rzeszow, Zelwerowicza 4, 35-601 Rzeszow, Poland

**Keywords:** Acrolein, Allyl alcohol, Apoptosis, Cell death, Oxidative stress, Superoxide dismutase, Yeast

## Abstract

The yeast *Saccharomyces cerevisiae* is a useful eukaryotic model to study the toxicity of acrolein, an important environmental toxin and endogenous product of lipid peroxidation. The study was aimed at elucidation of the cytotoxic effect of acrolein on the yeast deficient in SOD1, Cu, Zn-superoxide dismutase which is hypersensitive to aldehydes. Acrolein generated within the cell from its precursor allyl alcohol caused growth arrest and cell death of the yeast cells. The growth inhibition involved an increase in production of reactive oxygen species and high level of protein carbonylation. DNA condensation and fragmentation, exposition of phosphatidylserine at the cell surface as well as decreased dynamic of actin microfilaments and mitochondria disintegration point to the induction of apoptotic-type cell death besides necrotic cell death.

## Introduction

Acrolein is an environmental and intracellular-formed toxin. This compound is generated in many industrial processes like synthesis of organic compounds and is a product of combustion, smoking, automotive exhaust and is also formed endogenously as a lipid peroxidation product [1]. The toxicity of acrolein is involved in etiology of many devastating disorders including human neurodegenerative diseases. Understanding the cellular and molecular effects of acrolein would contribute to efficient attenuation of its toxicity. It has been shown by us that allyl alcohol may be used as a metabolic precursor in studies of acrolein toxicity [2]. Allyl alcohol is oxidized in the cells by alcohol dehydrogenase forming an aldehyde acrolein. The use of a precursor of acrolein instead of this highly reactive and unstable aldehyde allows avoiding the unwelcome reactions. We reported recently that the toxicity of acrolein generated from allyl alcohol in the yeast *Saccharomyces cerevisiae* cells involves oxidative stress as judged by glutathione (GSH) depletion, protection by low-molecular antioxidants and hypoxic atmosphere, induction of lipid peroxidation and Yap1p activation [3]. However, various models have been used to study the effects of reactive aldehydes, mainly 4-hydroxy-2-nonenal (HNE), including human cell lines [4], mammalian cells, and organs [5], fish [6], green algae [7], the yeast *Saccharomyces cerevisiae* appears an excellent model for studying the toxicity of exogenous reactive aldehydes because yeast cells do not produce ω-6 polyunsaturated fatty acids and thus are not susceptible to lipid peroxidation [8]. Yeast cells can however absorb the polyunsaturated fatty acids from the medium if present, and incorporate to cellular lipids [9]. The studied exogenous reactive aldehydes in yeast are thus not influenced by endogenous lipid peroxidation products.

To further elucidate the mechanism of acrolein toxicity to yeast cells, we studied the effects of allyl alcohol treatment on the yeast cells viability comparing to the effects of hydrogen peroxide and menadione, the commonly used toxicants inducing oxidative stress and cell death. Exogenous H_2_O_2_ was the first compound shown to trigger apoptosis in yeast cells and is the classical stimulus commonly used to induce yeast apoptosis [10, 11]. On the contrary to H_2_O_2_ which is a direct oxidant, menadione (2-methyl-1,4-naphthoquinone, vitamin K3) is a pro-oxidant drug. Cytotoxicity of menadione results from generating reactive oxygen species (ROS) in redox cycling of semiquinone radicals generated by enzymatic one-electron reduction of menadione and from electrophilic abilities to react with thiol groups of the proteins and GSH [12]. Menadione was shown to induce cell death through apoptosis in Jurkat cells [13], pancreatic acinar cells [14], and yeast cells [15].

The aim of this paper was to get further insight into the mechanism of the cytotoxic effect of acrolein on the yeast. We focused on the question whether the toxicity of acrolein generated from allyl alcohol for yeast cells results from growth arrest or leads to cell death. We used ∆*sod1* cells which were found previously as hypersensitive to acrolein [2]. The knock-out of *SOD1* gene encoding SOD1, Cu, Zn-superoxide dismutase, a crucial enzyme in removing superoxide anion in the cytosol, entails the hypersensitivity to a variety of stress agents due to escalated oxidative stress [16].

We show that allyl alcohol treatment causes oxidative stress by increasing secondary ROS production, increasing the level of protein carbonyls, and causes metabolic changes triggering cell death including actin depolymerization, loss of mitochondrial potential, and decrease of metabolic activity. The mode of cell death induced by allyl alcohol exhibits features of apoptosis-like DNA degradation, chromatin condensation, and phosphatidylserine exposure.

## Materials and Methods

### Chemicals

Allyl alcohol, CAS number 107-18-6, ≥99 %, was from Aldrich (Sigma-Aldrich, Poznan, Poland). 4′,6-diamidyno-2-fenyloindol, dihydroethidine, FUN-1, MitoTrackerGreen FM, rhodamine B hexyl ester and rhodamine−phalloidin stains were from Molecular Probes (Eugene, OR, USA). In Situ Cell Death Detection Kit, fluorescein (terminal deoxynucleotidyl transferase dUTP nick end labeling, TUNEL test) was from Roche (Roche Applied Science, Mannheim, Germany). Annexin V and propidium iodide were from Biotium (Hayward, CA, USA). Components of culture media were from DB Difco (Becton–Dickinson and Company, Spark, USA), except for glucose (POCh, Gliwice, Poland). All other reagents were purchased from Sigma-Aldrich (Poznan, Poland).

### Yeast Strains, Media, and Growth Conditions

The following yeast strains were used: wild-type SP4 MATα leu1 arg4 [17], and Δ*sod1* mutant, isogenic to SP4, MATα leu1 arg4 sod1::natMX [18]. Yeast was grown in a standard liquid YPD medium (1 % Yeast Extract, 1 % Yeast Bacto-Peptone, 2 % glucose) on a rotary shaker at 150 rpm or on a solid YPD medium containing 2 % agar, at a temperature of 28 °C. Cells from exponential phase culture (~16 h) were centrifuged, washed twice, suspended to a final density of 10^8^ cells/ml in 100 mM phosphate buffer, pH 7.0, containing 1 mM EDTA and 0.1 % glucose, and incubated at 28 °C with shaking for 60 min with 10 mM H_2_O_2_, 0.105 mM menadione or 0.4 mM allyl alcohol. Control cells were incubated for 60 min without or with the addition of ethanol (menadione solvent). Ethanol at the concentration used in the experiments did not affect the growth of the yeast cells and tested parameters (not shown). After incubation, the cells were centrifuged, washed twice, and used for further analysis.

### Toxicity Assays

For spotting tests, the cells after incubation were diluted to 10^7^, 10^6^, 10^5^, or 10^4^ cells/ml. Aliquots (5 µl) of each suspension were inoculated on solid YPD medium containing 2 % agar. Cells growth was inspected after 48 h. For verification of the cells budding, 5 µl of the cell suspensions were spotted on the plate with solid YPD medium and the pictures of the cells were taken using the Olympus BX-50 microscope equipped with Sony SSP-DC50AP digital camera at the beginning of the experiment and after 24 h.

### Assessment of Metabolic Activity of the Cells

After incubation the cells were suspended in 10 mM Na-HEPES, pH 7.2, containing 2 % glucose. Metabolic activity of cells was estimated with 1 µM FUN-1 stain (100 μM stock in DMSO). Metabolically active cells contain cylindrical, red fluorescent structures in their vacuoles; little or no metabolic active cells have diffuse green cytoplasmic fluorescence and lack fluorescent intravacuolar bodies, while dead cells exhibit extremely bright, diffuse, green-yellow fluorescence [19]. Metabolic activity of the cells was expressed as a change in ratio of red (*λ* = 575 nm) to green (*λ* = 535 nm) fluorescence. The fluorescence of the cell suspensions was measured after 30 min since addition of FUN-1 using Tecan Infinite M200 microplate reader at *λ*_ex_ = 480 nm, *λ*_em_ = 500–650 nm.

### ROS Generation Assay

Generation of reactive oxygen species was assessed with dihydroethidine (DHET; 18.9 µM final concentration; 3.17 mM stock in DMSO) [20]. Cells from exponential phase culture were centrifuged, washed twice, and suspended to the density of 10^8^ cells/ml in 100 mM sodium phosphate buffer, pH 7.0, containing 1 mM EDTA and 0.1 % glucose. The cell suspensions were added with 10 mM H_2_O_2_, 0.105 mM menadione, or 0.4 mM allyl alcohol concurrently with DHET or were incubated with 10 mM H_2_O_2_, 0.105 mM menadione, or 0.4 mM allyl alcohol for 60 min, centrifuged, washed twice, resuspended in the buffer and then added with DHET. The kinetics of fluorescence increase, due to oxidation of the fluorogenic probe, was measured immediately after addition of DHET using the Hitachi F2500 fluorescence spectrophotometer at *λ*_ex_ = 518 nm and *λ*_em_ = 605 nm at the temperature 28 °C. There were no cross-reactions between the probe and tested chemicals in a blank—the buffer without the cells (not shown).

### Determination of Protein Carbonylation

After incubation with 10 mM H_2_O_2_, 0.105 mM menadione, or 0.4 mM allyl alcohol, the cells were suspended in protein extraction buffer (PEB) containing 10 % glycerol, 2 mM EDTA, 1 mM PMSF, and MilliQ water. The cells were disrupted with 0.5 mm glass beads in 5 cycles of 30 s with intervals for cooling the sample in ice and centrifuged (14,000×*g*, 15 min, 4 °C). The supernatants were used for the assay. Protein concentration was determined using the Bradford method. The content of carbonyl groups in the protein samples was determined by reaction with 2,4-dinitrophenylhydrazine (DNPH) and detected with anti-DNP antibodies according to Levine et al. [21]. The DNP-derivatized proteins (7.5 µg/lane) were separated by SDS-PAGE. Next the proteins were transferred to nitrocellulose membrane (PVDF Western Blotting Membranes, Roche) by semidry immunoblotting (Bio-Rad Laboratories, Inc) at 100 V for 60 min, at 4 °C. After blocking with PBST buffer (PBS, 0.1 % Tween 20) containing 3 % nonfat milk, the membrane was incubated with the primary polyclonal rabbit antibody specific to the DNP moiety of the proteins (ab6306, Abcam), at a 1:20,000 dilution. After 180 min incubation, the membrane was washed and subsequently incubated with the secondary goat anti-rabbit antibodies conjugated with HRP (111,035,003, Jackson Immuno Research) at a 1:20,000 dilution. Immunodetection was performed by chemiluminescence using a SuperSignal West PICO Chemiluminescent Substrate (Pierce Biotechnology) according to the manufacturer’s instructions, and the images were captured using a BioSpectrum^®^ Image System (Ultra-Violet Products Ltd.). Quantitative analysis of chemiluminescence signal was performed by densitometry using the VisionWorks LS software.

### Cell Viability Assays

After incubation with 10 mM H_2_O_2_, 0.105 mM menadione, or 0.4 mM allyl alcohol, the cells were suspended in phosphate-buffered saline (PBS). The viability of the cells was estimated by co-staining with 5 µg/ml propidium iodide (PI; 1 mg/ml stock in MilliQ water) and 10 µg/ml fluorescein diacetate (FDA; 1 mg/ml stock in acetone) [22] and also by staining with 10 μg/ml Phloxine B (2 mg/ml stock in MilliQ water) [23]. FDA/PI fluorescence was examined at *λ*_ex_ = 480 nm under the Olympus BX-51 microscope equipped with the DP-72 digital camera. The viable cells were green fluorescent and the dead cells were red fluorescent. The results were shown as a number of PI-positive cells. Red, phloxine B-positive cells (dead cells) were examined under bright field microscope Olympus BX-51 equipped with a DP-72 digital camera. The experiment was performed in 3 independent biological replicates, in each replicate at least 200 cells were analyzed.

### Detection of Apoptotic Phenotype

#### DAPI Nuclei Staining

Yeast cells nuclei were stained with 4′,6-diamidino-2-phenylindole (DAPI). After incubation with 10 mM H_2_O_2_, 0.105 mM menadione, or 0.4 mM allyl alcohol, the cells were harvested, washed twice with PBS buffer, and resuspended in PBS buffer. DAPI was added to a final concentration 2 μg/ml (100 μg/ml stock solution in MilliQ water). After 10 min of incubation, the morphology of the cell nuclei were observed using a fluorescence microscopy at *λ*_ex_ = 360 nm and *λ*_em_ = 420 nm. Apoptotic nuclei were identified by a condensed chromatin or nuclei fragmentation to form the nuclear bodies.

#### TUNEL Assay

DNA strand breaks were monitored by terminal deoxynucleotidyl transferase dUTP nick end labeling (TUNEL) with the In Situ Cell Death Detection kit, Fluorescein as described by [10]. After treatment with H_2_O_2_, menadione or allyl alcohol yeast cells were washed twice with PBS buffer and fixed in PBS buffer with 3.7 % (vol/vol) formaldehyde for 30 min at room temperature. The fixed cells were washed three times with PBS buffer and then the cell wall was digested with 15 U/ml lyticase for 60 min at 37 °C. The cell suspension was then applied to polylysine-coated slides and dried for 30 min at 37 °C. The slides were rinsed with PBS buffer, incubated in permeabilization solution (0.1 % Triton X-100 and 0.1 % sodium citrate) for 2 min on ice, rinsed twice with PBS buffer, and incubated for 60 min at 37 °C in a humidified box with 10 μl of TUNEL reaction mixture prepared according to the manufacturer’s protocols. The slides were then rinsed three times with PBS buffer and observed under fluorescence microscope. The fluorescence was examined at *λ*_ex_ = 490 nm and *λ*_em_ = 515 nm.

#### Annexin V and Propidium Iodide Staining

Exposed phosphatidylserine (PS) was detected by CF488A conjugated Annexin V (Ann V). After incubation with H_2_O_2_, menadione, or allyl alcohol, the cells were harvested and washed twice with sorbitol buffer (1.2 M sorbitol, 0.5 mM MgCl_2_, 53 mM potassium phosphate, pH 6.8), resuspended in sorbitol buffer, then digested with 15 U/ml lyticase for 30 min at 30 °C. Cells were then harvested, washed twice in binding buffer (10 mM HEPES, 140 mM NaCl, 2.5 mM CaCl_2_, 1.2 M sorbitol, pH 7.4) and resuspended in 50 μl binding buffer. 2 μl of Annexin V-CA488 and 2 μl of propidium iodide (PI; 500 μg/ml) were added to 38 μl cell suspension and incubated for 30 min at room temperature in the dark. The cells were harvested, suspended in binding buffer, and applied to a microscopic slide. Annexin V with CF488A enables to identify apoptotic cells (green fluorescence) by binding to cell surfaces exposed to phosphatidylserine. Co-staining with propidium iodide, a non-cell-permeable DNA dye, indicates necrotic cells (red fluorescence). Annexin V/PI fluorescence was examined using fluorescence microscope at *λ*_ex_ = 490 nm and *λ*_em_ = 515 nm.

#### *Rhodamine*−*Phalloidin Staining*

Actin cytoskeleton was stained with rhodamine−phalloidin. After incubation with H_2_O_2_, menadione, or allyl alcohol, the cells were harvested, washed twice with PBS buffer, and fixed in PBS buffer with 4 % formaldehyde for 60 min. The fixed cells were washed three times with PBS buffer and resuspended in 1 ml PBS. 16 μl of rhodamine−phalloidin (300 U/ml stock in methanol) were added to 100 μl of cell suspension and incubated for 60 min at room temperature in the dark. The cells were harvested, suspended in glycerol-based mounting medium, and applied to a microscopic slide. Fluorescence was examined at *λ*_ex_ = 490 nm and *λ*_em_ = 516 nm.

#### Mitochondria Visualization

Mitochondria were stained with MitoTrackerGreen FM—a green-fluorescent mitochondrial stain, which localizes in mitochondria regardless of mitochondrial membrane potential and rhodamine B hexyl ester—a red fluorescent stain, which localizes in mitochondria depending of mitochondrial membrane potential. The cells after treatment with H_2_O_2_, menadione, or allyl alcohol were washed twice with PBS buffer and resuspended in 10 mM HEPES buffer, pH 7.4, containing 5 % glucose. MitoTrackerGreen (10 μM stock solution in DMSO) was added to a final concentration 100 nM. After 15 min of incubation, mitochondria were visualized by fluorescence microscopy at *λ*_ex_ = 490 nm and *λ*_em_ = 516 nm. Rhodamine B (10 μM stock solution in DMSO) was added to a final concentration of 100 nM. After 15 min of incubation, mitochondrial membrane potential was visualized by fluorescence microscopy at *λ*_ex_ = 555 nm and *λ*_em_ = 579 nm.

All observations were carried out using the Olympus BX-51 epifluorescence microscope equipped with the DP-72 digital camera, Cell^D software. Each experiment was performed in 3 independent biological replicates, in each replicate at least 150 cells were analyzed.

### Statistical Analysis

Data are presented as mean ± SD from at least 3 independent experiments or consistent results of a typical experiment reproduced at least three times. Statistical analysis was performed using STATISTICA 10 software (StatSoft, Inc.). The statistical significance of differences between means of treated samples compared to untreated control was estimated using one-way ANOVA and the Dunnett post hoc test. The differences between means of two yeast strains compared were evaluated using the *t* test for independent samples. Values were considered significant if *P* < 0.05.

## Results

### Acrolein Hampers the Yeast Growth

**Fig. 1:**
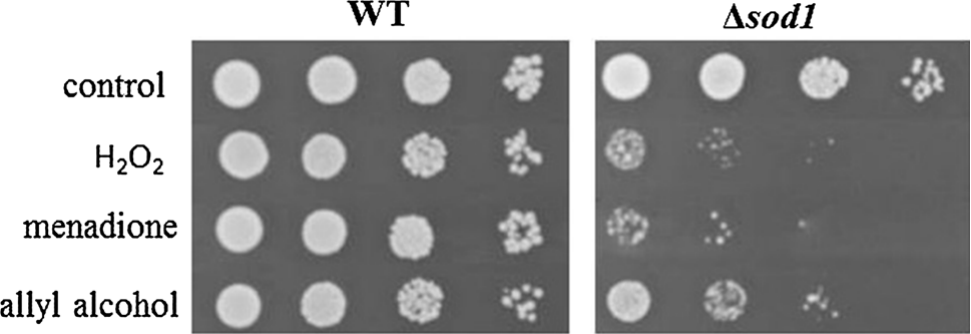
The effects of hydrogen peroxide, menadione, and allyl alcohol treatment on the growth of yeast cells. The cells were treated with 10 mM H_2_O_2_, 0.105 mM menadione, or 0.4 mM allyl alcohol for 1 h and grown on YPD plate. Each drop contained sequentially 50,000, 5,000, 500, and 50 cells. The growth was inspected after 48 h

**Fig. 2:**
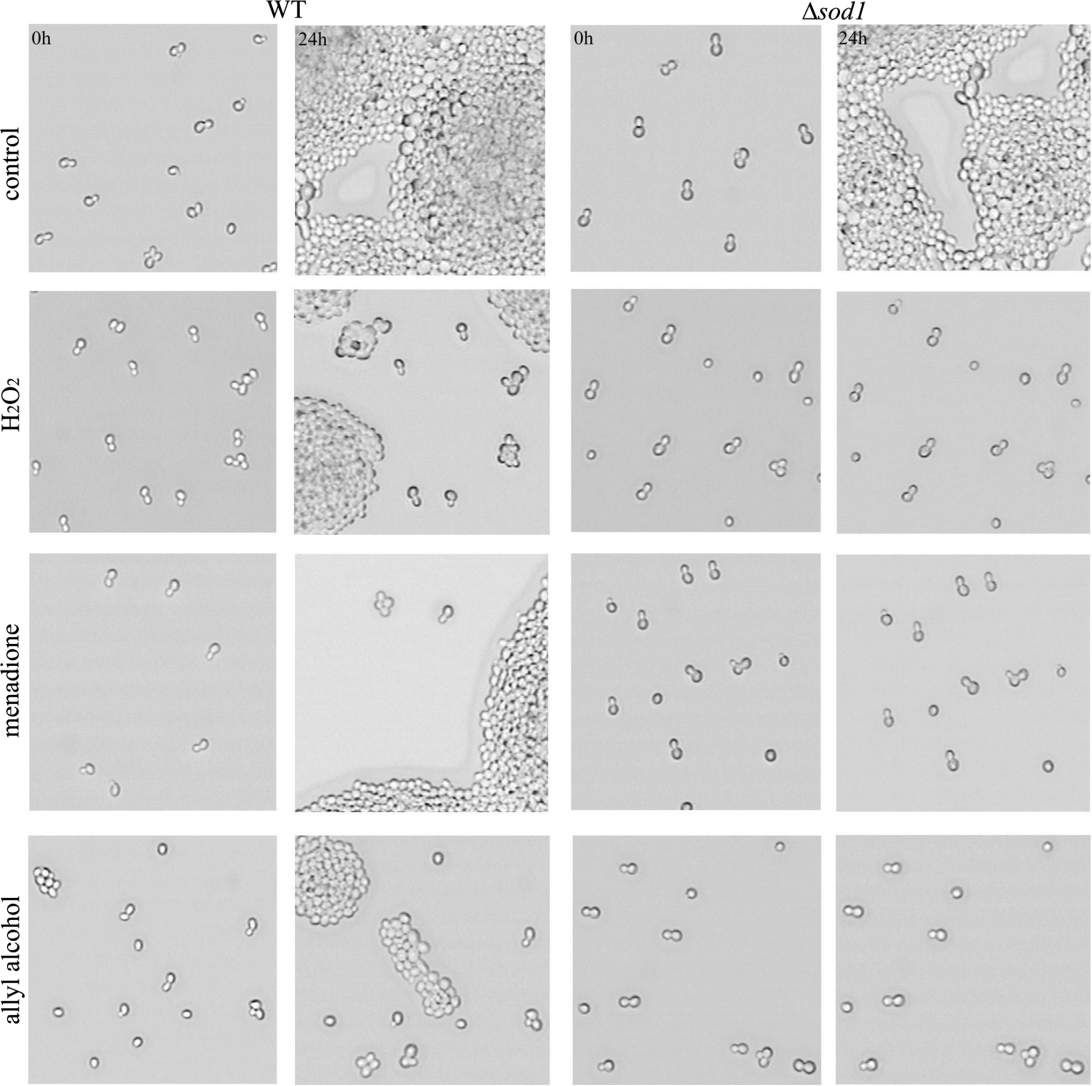
The effects of hydrogen peroxide, menadione, and allyl alcohol treatment on the budding ability of yeast cells. The cells were treated with 10 mM H_2_O_2_, 0.105 mM menadione, or 0.4 mM allyl alcohol for 1 h and spread on YPD plate. The cells budding was monitored under the microscope

We examined the effects of acrolein generated from allyl alcohol on wild-type and ∆*sod1* cells in comparison to hydrogen peroxide and menadione. For detailed study of the toxicity of acrolein in the cells, we used the dose of these toxicants which partially inhibited the growth of the mutant strain after 1 h treatment as assessed in drop test (Fig. 1). The concentrations were as follows: 10 mM H_2_O_2_, 0.105 mM menadione, and 0.4 mM allyl alcohol. These doses caused the inhibition of ∆*sod1* cells budding and colonies formation but not wild-type strain (Fig. 2).

### Acrolein Induces Oxidative Stress

The involvement of oxidative stress arising from the loss of glutathione in the toxicity of acrolein formed from allyl alcohol was shown by us previously [3]. To further evaluate intracellular perturbations conducting the growth inhibition observed after treatment the cells with allyl alcohol, we examined the metabolic activity, ROS production, and protein carbonyls content. The metabolic activity was estimated with FUN-1 stain. The higher red/green fluorescence ratio, the higher metabolic activity demonstrated by the cells [19].

**Fig. 3:**
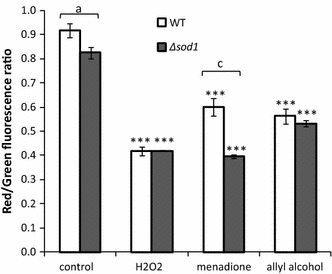
Metabolic activity of the cells treated with hydrogen peroxide, menadione, and allyl alcohol estimated with FUN-1 stain. The cells after treatment with 10 mM H_2_O_2_, 0.105 mM menadione, or 0.4 mM allyl alcohol were added with FUN-1 and the fluorescence was examined. Data are expressed as ratio of red (*λ* = 575 nm) to green (*λ* = 535 nm) fluorescence and presented as mean ± SD from 3 independent experiments. ***P < 0.001 of significant different values with respect to untreated control within the same yeast strain estimated with ANOVA and Dunnett post hoc test. The letters ^a, b, c^ indicate significant differences at *P* < 0.05, *P* < 0.01, and *P* < 0.001, respectively, between wild-type and mutant strain estimated with *t* test for independent samples. Where no *error bar* is indicated, it was too small to be visible

The cells exposed to hydrogen peroxide, menadione, and allyl alcohol exhibited impaired metabolic activity estimated with FUN-1 stain in case of both strains (Fig. 3). Metabolic activity of ∆*sod1* untreated cells was significantly lower than wild-type strain and also after menadione treatment was decreased markedly as compared to wild-type strain (Fig. 3).

**Fig. 4:**
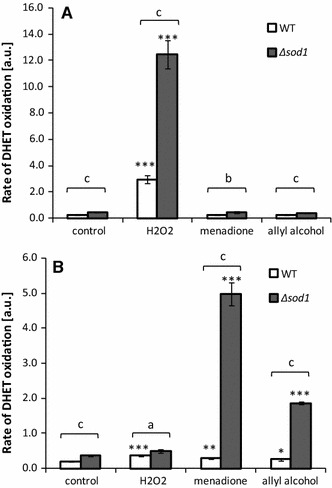
ROS production in the cells estimated with dihydroethidine **a** immediately after addition of hydrogen peroxide, menadione, or allyl alcohol; **b** 1 h after treatment with hydrogen peroxide, menadione, and allyl alcohol. *P < 0.05, **P < 0.01, and ***P < 0.001 of significant different values with respect to untreated control within the same yeast strain estimated ANOVA and Dunnett post hoc test. The letters ^a,b,c^ indicate significant differences at *P* < 0.05, *P* < 0.01, and *P* < 0.001 respectively comparing wild-type and mutant strain estimated with *t* test for independent samples. Where no *error bar* is indicated, it was too small to be visible

We estimated ROS level after treatment with hydrogen peroxide, menadione, and allyl alcohol. In order to catch the possible differences between the effects of hydrogen peroxide which is a reactive oxygen species and the substrate of antioxidant enzyme catalase and of menadione and allyl alcohol which must be metabolized enzymatically to attend redox cycling or produce acrolein, respectively, we measured ROS content directly after addition of the studied chemicals (i.e., within 30 min as the ROS content was estimated as the rate of fluorescence increase) and after 1 h. ROS generation was significantly higher in ∆*sod1* cells than in wild-type and was elevated by treatment with the chemicals studied but only after incubation (Fig. 4b). Assessment of ROS generation immediately after addition of H_2_O_2_, menadione, and allyl alcohol did not show increased ROS production except for hydrogen peroxide (Fig. 4a). This is consistent with our previous results which indicated no alterations in ROS generation in the cells directly after addition with allyl alcohol [3].

**Fig. 5:**
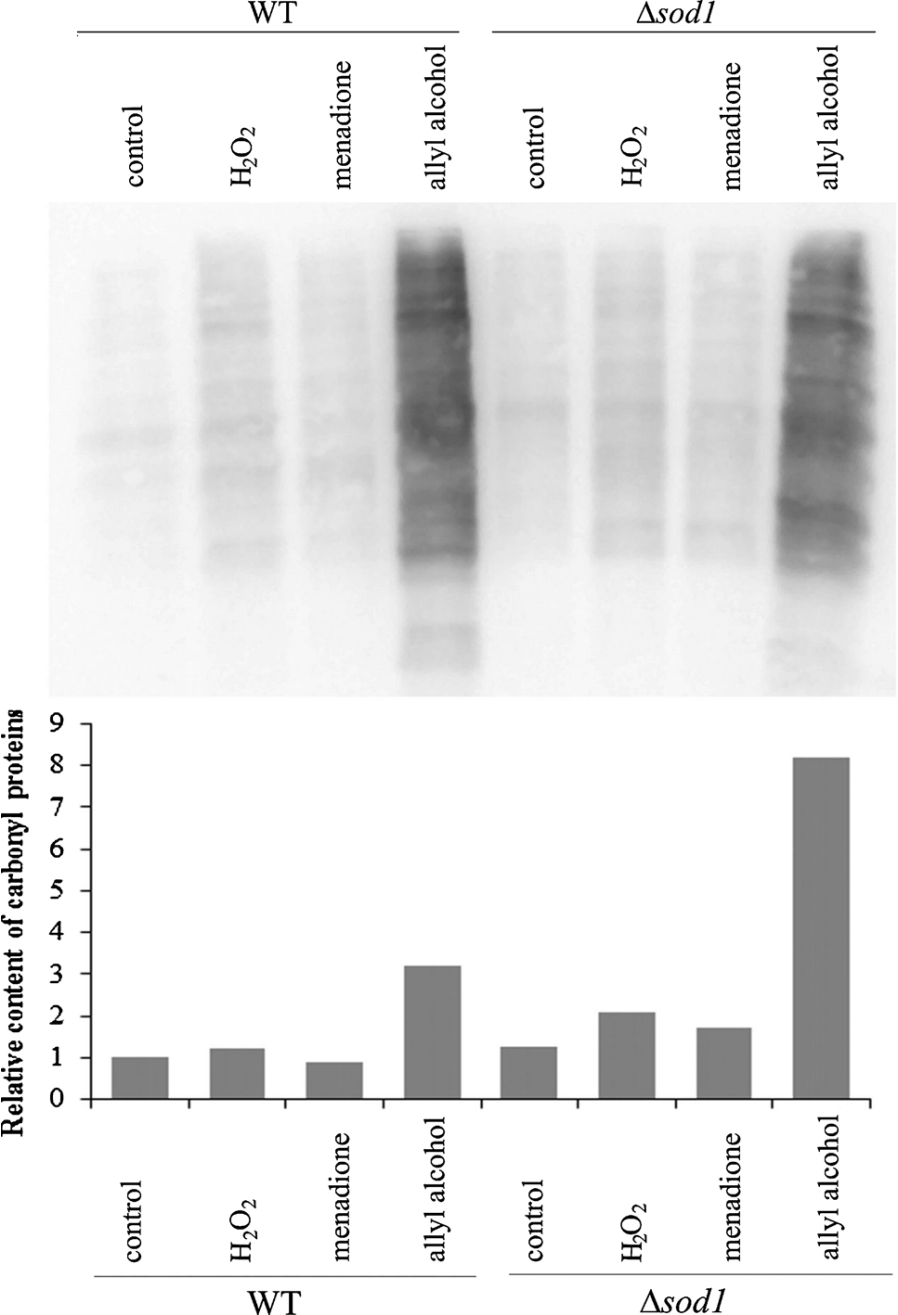
Protein carbonylation in yeast cells treated with hydrogen peroxide, menadione, or allyl alcohol. The content of carbonyl groups in the protein samples was determined by reaction with 2,4-dinitrophenylhydrazine (DNPH) and detected with anti-DNP antibodies. Data present the typical result of duplicate experiment

To find if the growth inhibition was accompanied by accumulation of protein damage, we assessed the content of protein carbonyls after 1 h treatment with H_2_O_2_, menadione, and allyl alcohol. Figure 5 shows the level of carbonylated proteins in wild-type and ∆*sod1* strains. The enormous increase in protein carbonyls was induced in the cells exposed to allyl alcohol, notably in ∆*sod1* strain (several fold as compared to untreated control). The effect of hydrogen peroxide and menadione was not so marked in ∆*sod1* strain and negligible in wild-type cells (Fig. 5).

### Acrolein Induces Cell Death

**Fig. 6:**
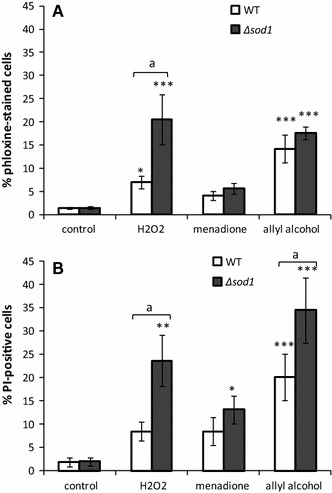
The viability of the cells exposed to hydrogen peroxide, menadione, or allyl alcohol estimated **a** with phloxine B as % of dead cells, and **b** with FDA/PI co-staining as % of dead cells. The cells were treated with 10 mM H_2_O_2_, 0.105 mM menadione, or 0.4 mM allyl alcohol, added with phloxine B or FDA and PI and the stained cells were observed and counted using microscope. *P < 0.05,** *P* < 0.01, and ***P < 0.001 of significant different values from untreated control within the same yeast strain using ANOVA and Dunnett post hoc test. The letter ^a^ indicates significant differences at *P* < 0.05 comparing wild-type and mutant strain with *t* test for independent samples

For further examination, the causations of colonies formation inhibition in Δ*sod1* cells by treatment with allyl alcohol, the cells were stained with phloxine B or propidium iodide, the dyes which stains the dead cells only. Under these conditions (1 h treatment, 50 % growth inhibition in the drop test), most of the cells were viable and not able to divide but the number of dead cells reached ~20 % in Δ*sod1* strain after H_2_O_2_ treatment and ~15–20 % in wild-type and Δ*sod1* strains after treatment with allyl alcohol (Fig. 6a).

Co-staining the cells with FDA and PI after treatment with tested chemicals revealed similar results; however, the number of dead cells was greater than with phloxine B staining (Fig. 6b).

Dead, PI-positive cells are necrotic or late-apoptotic [24]. Interestingly, the difference between the number of necrotic−dead cells estimated with phloxine B and the number of necrotic or late-apoptotic cells estimated as PI-positive was largest in Δ*sod1* cells treated with allyl alcohol suggesting that acrolein may trigger apoptosis.

**Fig. 7:**
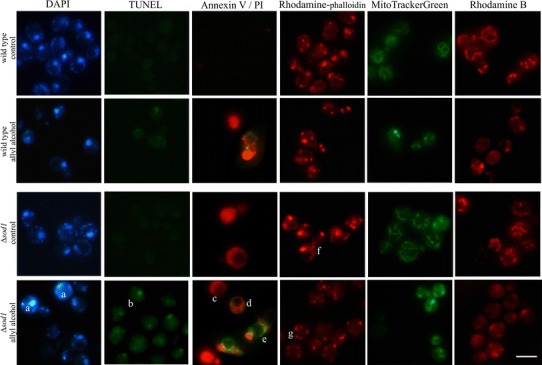
Visualization of apoptotic markers and organelles structures in yeast cells after treatment with allyl alcohol. Data are shown as representative microphotographs of the cells treated with 0.4 mM allyl alcohol, stained with appropriate fluorescent dyes (DAPI, TUNEL, Annexin V/PI for detection typical apoptotic hallmarks, rhodamine−phalloidin for actin cytoskeleton, MitoTrackerGreen for mitochondria structure, rhodamine B for mitochondrial membrane potential), and observed under fluorescent microscope. The signs indicate *a* chromatin condensation estimated with DAPI, *b* TUNEL^+^ cells, apoptotic DNA degradation, *c* Ann^−^, PI^+^ cells, necrotic-dead cells with ruptured plasma membrane, *d* Ann^+^, PI^+^ cells, late-apoptotic, or secondary necrotic cells, *e* Ann^+^, PI^−^ cells, apoptotic dead cells, *f* actin cables, *g* actin patches. The *bar* denotes 5 µm (Color figure online)

To discriminate the mode of cell death, we examined the hallmarks of apoptosis. Figure 7 shows the effects of acrolein generated from allyl alcohol on typical apoptotic markers like DNA integrity, PS exposition, and also actin cytoskeleton and mitochondria architecture in the treated cells. DAPI stained cells of ∆*sod1* strain show apoptotic phenotype after treatment with allyl alcohol as chromatin condensation (Fig. 7). The significant increase in the number of TUNEL-positive cells is observed in ∆*sod1* cells and slight in wild-type cells, indicating apoptotic DNA fragmentation (Fig. 7). This correlates with the observation of the externalization of phosphatidylserine in ∆*sod1* cells (AnnV^+^ cells) which is considered as an undoubted marker of occurrence of apoptosis [24]. A large diversity in the mode of cell death in ∆*sod1* cells caused by acrolein was revealed by Annexin V staining. Co-staining the cells with Annexin V and PI shows the necrotic red fluorescent cells (AnnV^−^, PI^+^), late-apoptotic, or secondary necrotic red/green fluorescent cells (AnnV^+^, PI^+^) exhibiting as well as PI fluorescence due to ruptured plasma membrane and Annexin V fluorescence due to exposure of PS, and green fluorescent early apoptotic cells which show only PS exposition. We observed also the alterations in cytoskeleton organization caused by allyl alcohol treatment in ∆*sod1* cells (Fig. 7) which are ascribed to apoptotic cell death [25]. Actin cytoskeleton is assembled in two structures—cables and patches. Actin cables are highly dynamic structures and serve as the tracks for organelle movements including mitochondria and vacuoles. Allyl alcohol treatment caused actin destabilization estimated as rhodamine−phalloidine stained actin patches only but not cables. This effect was much more pronounced in ∆*sod1* cells. Allyl alcohol treatment implicated also mitochondrial network fragmentation as well as the loss of mitochondrial membrane potential (Fig. 7). These effects were again much more evident in ∆*sod1* cells than in wild-type.

## Discussion

Acrolein, an unsaturated aldehyde generated in smoking, combustion, food processing, biological, and industrial processes became a subject of investigations in view of its reactivity. Acrolein is the strongest electrophile among reactive aldehydes [26]. There is a growing evidence of the connection of acrolein toxicity and the human pathologies e.g., Alzheimer's disease, spinal cord injury, and others [27–29]. Toward to this, the elucidation of the toxic effects of acrolein on the cells viability and the mode of cell death appears particularly important.

The yeast *Saccharomyces cerevisiae* is thought unquestionably as a valuable tool to elucidate major cellular processes in Eukaryotes as well as the mechanisms of programmed cell death. Although the link between oxidative stress and programmed cell death seems to be well established, the involvement of oxidative stress and thus the possible mechanisms of cell death caused by various environmental stimuli or drugs are still under evaluation. Till now, various external stimuli has been found to induce apoptosis in yeast i.e., hydrogen peroxide, acetic acid, ethanol, hypochlorous acid, hyperosmolarity, heat stress, metals as well as pharmacological agents like aspirin, amiodarone, and others [24].

It was shown previously that yeast cells deficient in Cu, Zn-superoxide dismutase (SOD1) are hypersensitive to acrolein. Using this mutant strain to study acrolein toxicity seems a promising model in view of the involvement of lipid peroxidation products in pathology of neurodegenerative disorders, and on the other hand, the decreased SOD activity in neurodegenerative diseases [28–31].

Cu, Zn-superoxide dismutase is the isoform of enzyme removing superoxide anion, localized in the cytosol and mitochondrial intermembrane space [32]. The lack of this protein results in the hypersensitivity of the cells to many oxidants, such as *t*-butyl hydroperoxide, cumene hydroperoxide, 2,2-azobis-(2-amidinopropane) (AAPH), chloramines, menadione, juglone, oxytetracycline [33], hypertonic stress [18], paraquat, diquat, plumbagin, benzoquinone, H_2_O_2_ [34], peroxynitrite and nitric oxide [35], or hypochlorite and chlorite [36].

In this work, we show that treatment of the yeast ∆*sod1* cells with acrolein generated from allyl alcohol implies oxidative stress and causes cell death. The same effect of hydrogen peroxide, menadione, and acrolein in the form of partial reduction of colony formation abilities (Fig. 1) arose from similar metabolic alterations. Growth inhibition caused by these chemicals was evoked by the decrease in metabolic activity (Fig. 3) which correlated with increased ROS generation and accumulating of damaged proteins (Figs. 4, 5). As compared to the effects of hydrogen peroxide and menadione, acrolein-induced ROS production was not such elevated.

Hydrogen peroxide as direct oxidant increased markedly the intracellular ROS level immediately after addition, and ROS content was reduced again after 60 min remaining similar to the control in ∆*sod1* cells and higher than control in wild-type cells but still significantly lower than in mutant cells. H_2_O_2_ is the classical inducer of Yap1-dependent oxidative stress response mediated by Gpx3. The transient ROS increase indicates that the response to oxidative stress generated by hydrogen peroxide is rapid (~30 min Yap1 activation after 1 mM H_2_O_2_ treatment) in yeast cells, as it has been shown earlier [37]. In contrast to hydrogen peroxide, menadione, and allyl alcohol did not induce ROS production immediately after addition. The increase in ROS production was significant in ∆*sod1* cells after 1 h incubation and much higher for menadione than for allyl acohol. Menadione as well as acrolein are also able to induce stress response via Yap1 transcription factor but the kinetic of this Gpx3-independent reaction typical for thiol-reactive agents is not such rapid [3, 38].

The major pathway of acrolein detoxification in the cells is conjunction of acrolein with glutathione. Acrolein may react also with other thiol-containing compounds like cysteine, 2-mercaptoethanesulfonate, 2,6-dithiopurine [39]. A rapid, dose-dependent decrease in glutathione content in the cells treated with allyl alcohol was shown previously by us [2, 3]. The predominant reactivity of acrolein with GSH, and hence disturbance of cellular redox buffer, may be the cause of increased ROS production in the cells, especially ∆*sod1* mutant.

It has been reported that ROS may be generated by enzymatic metabolism of acrolein by aldehyde dehydrogenase or by acrolein metabolite—glutathionylpropionaldehyde a product of acrolein and GSH conjunction [40]. However in yeast cells although this reaction is possible, its impact seems rather inconsiderable as the expression of two cytosolic forms of aldehyde dehydrogenase (*ALD2*, *ALD3*) is repressed on glucose medium, only *ALD6* is constitutively expressed among cytosolic isoforms [41]. The ROS production induced by acrolein in yeast cells, as seen by comparison with the effects of menadione and hydrogen peroxide, appears to be a secondary result of thiol depletion.

Increased ROS production evoked by H_2_O_2_, menadione, and allyl alcohol correlated with elevated content of protein carbonyls. In contrast to the effects of menadione and hydrogen peroxide which induced slight increase in damaged proteins content, allyl alcohol treatment caused enormous accumulation of carbonylated proteins (Fig. 5). It is appointed that low oxidative stress causes oxidation of protein -SH groups to form mixed disulfides which are back reduced when the oxidative stress is diminished or degraded by proteasomes. Strong oxidative stress involves protein oxidative modifications which cannot be repaired, they may alter protein structure and functions, and it may lead to accumulation of damaged proteins what in consequence conduces to cellular perturbations. Acrolein and other reactive aldehydes may covalently modify the proteins by formation adducts with amino acids by Schiff base formation or Michael addition. Among amino acids, cysteine, lysine, and histidine have the highest reactivity with aldehydes [1]. Many proteins were found to be modified by acrolein, both cytosolic (identified in human cells) and mitochondrial (identified in rat cardiac mitochondria) [42]. The increased content of carbonylated proteins in yeast cells treated directly with acrolein at much higher concentration (1 and 2 mM) in comparison with using allyl alcohol as acrolein precursor (0.4 mM) was shown previously [43].

The excessive increase in carbonyl protein content caused by allyl alcohol treatment observed in our experiments, especially in ∆*sod1* strain, together with increased secondary ROS production, tended us to study if the acrolein exposure leads to cell death. We found that the growth arrest resulted partly from cell death as a number of dead cells was detected after application of the tested chemicals to the cells (Fig. 6). Phloxine B staining of dead cells in population indicated that in contrast to wild-type, ∆*sod1* mutant exhibited higher number of dead cells after treatment with hydrogen peroxide what is consistent with the results of drop test, while in case of menadione and allyl alcohol, the occurrence of dead cells seems similar when comparing the effect between the two strains. This suggests that the effect of growth inhibition of ∆*sod1* strain shown by drop test (Fig. 1) or microscopic observations of cell budding (Fig. 2) results mainly from growth arrest and inability to divide not from massive cell death. Similar results in the form of the yeast growth arrest were also found previously for 4-hydroxy-2-nonenal [44]. Staining the cells with PI revealed the higher number of dead cells than phloxine B staining suggesting the possibility of occurrence of apoptosis.

Necrosis was regarded an accidental, uncontrolled process of cell death with characteristic plasma membrane rupture, random DNA degradation, disintegration of cellular organelles. It has become clear nowadays that some necrotic events may also be under molecular control and thus may also be considered as programmed cell death [45, 46]. The same factors, e.g., hydrogen peroxide [10] and acetic acid [47] may be apoptotic or necrotic for the cells depending on the dose or other conditions. In our experimental conditions, the most similar number of necrotic-dead cells (phloxine B-positive) and necrotic/late-apoptotic cells (PI-positive) was found for hydrogen peroxide-treated cells (10 mM, 1 h) indicating prevalence of necrotic cell death. This is consistent with the observed excessive but transient ROS increase and the lesser extent of protein damage in comparison to allyl alcohol effect. In contrast to hydrogen peroxide, for menadione and allyl alcohol, the number of PI-positive cells was about twice higher than that of phloxine B-positive cells suggesting the possible occurrence of apoptosis. Menadione is well known as ROS generating and electrophile agent which may deplete glutathione pool [12, 48, 49]. This compound was also shown as a potential apoptosis inducer in Jurkat T-cells [13] or murine pancreatic acinar cells [14]. Our data also show increased ROS production and carbonyl proteins content by menadione treatment. Similar effects were observed for allyl alcohol treatment, although in comparison to menadione, the accumulation of carbonylated proteins seems much greater for allyl alcohol. Thus for acrolein, protein damage and GSH depletion appear as the predominant aspects of oxidative stress which might trigger apoptotic cell death. This prediction of occurring apoptosis was confirmed by typical apoptotic markers as occurrence of DNA condensation (DAPI staining) and fragmentation (TUNEL assay) or exposition of phosphatidylserine (Annexin V staining) (Fig. 7). Triggering apoptotic cell death involves also the alterations in cellular organelles i.e., cytoskeleton organization and mitochondria structure (Fig. 7). The alterations in actin cytoskeleton, especially the actin aggregation, may also tend to ROS accumulation because of destabilization of mitochondrial network [50, 51]. Actin remodeling plays an important role in response to stress, and a declined cytoskeleton dynamics may reduce the effectiveness of the stress response. Acrolein was reported to react covalently with amino acid residues in actin, preferentially with cysteine, but it did not influence actin depolymerization [52], indicating that the effect of reduced actin dynamics was rather indirect consequence of oxidative stress. Moreover, there is an association between actin organization and mitochondrial function. The actin cytoskeleton is involved in the movement of mitochondria along actin filaments and its dynamic state is also important for maintain the membrane potential of mitochondria [53]. Actin cytoskeleton alterations may induce cell death in yeast cells mediated through the changes in mitochondria such as mitochondria fragmentation and the reduced mitochondrial membrane potential. Fragmented and dysfunctional mitochondria are, however, associated with ROS accumulation and apoptotic cell death [54]. Induction of apoptotic cell death was also shown recently in cardiomyocytes [55] and oxidative stress and cytoskeletal changes in Sertoli cells [56].

In conclusion, our findings clearly show that acrolein induces oxidative stress in SOD1-deficient yeast cells by impairing metabolic activity, secondary ROS generation, protein modifications which in consequence lead to cell death including apoptosis. The apoptotic cell death induced by acrolein may probably occur in parallel with necrosis. Our findings elicit also the distinction between inducing cell death and the inhibition of yeast cell budding as the potential mechanisms of acrolein toxicity which in yeast may be incorrectly considered as the same when assessed by growth tests. The term “second messenger of free radicals” has been ascribed mainly to HNE [42, 57, 58], our data supports this idea also with respect to acrolein as it may interfere with proteins, modulate cell growth, cellular signaling, and trigger apoptosis.

## References

[1] Stevens JF, Maier CS (2008). Acrolein: Sources, metabolism, and biomolecular interactions relevant to human health and disease. Molecular Nutrition & Food Research.

[2] Bilinski T, Kwolek M, Sas E, Krynicka M, Koziol S, Owsiak-Teleon A, Krzepilko A, Bartosz G (2005). A novel test for identifying genes involved in aldehyde detoxification in the yeast. Increased sensitivity of superoxide-deficient yeast to aldehydes and their metabolic precursors. BioFactors.

[3] Kwolek-Mirek M, Bednarska S, Bartosz G, Biliński T (2009). Acrolein toxicity involves oxidative stress caused by glutathione depletion in the yeast *Saccharomyces cerevisiae*. Cell Biology and Toxicology.

[4] Chapple SJ, Cheng X, Mann GE (2013). Effects of 4-hydroxynonenal on vascular endothelial and smooth muscle cell redox signaling and function in health and disease. Redox Biology.

[5] Poli G, Schaur RJ, Siems WG, Leonarduzzi G (2008). 4-Hydroxynonenal: A membrane lipid oxidation product of medicinal interest. Medicinal Research Reviews.

[6] Bastos FF, Tobar SAL, Dantas RF, Silva ES, Nogueira NPA, Paes MC, Righi BDP, Cunha Bastos J, Cunha Bastos VLF (2013). Melatonin affects conjugation of 4-hydroxynonenal with glutathione in liver of pacu, a hypoxia-tolerant fish. Fish Physiology and Biochemistry.

[7] Spoljaric D, Cipak A, Horvatic J, Andrisic L, Waeg G, Zarkovic N, Jaganjac M (2011). Endogenous 4-hydroxy-2-nonenal in microalga *Chlorella kessleri* acts as a bioactive indicator of pollution with common herbicides and growth regulating factor of hormesis. Aquatic Toxicology.

[8] Cipak A, Hasslacher M, Tehlivets O, Collinson EJ, Zivkovic M, Matijevic T, Wonisch W, Waeg G, Dawes IW, Zarkovic N, Kohlwein SD (2006). Saccharomyces cerevisiae strain expressing a plant fatty acid desaturase produces polyunsaturated fatty acids and is susceptible to oxidative stress induced by lipid peroxidation. Free Radical Biology and Medicine.

[9] Mizoguchi H, Hara S (1997). Ethanol-induced alterations in lipid composition of Saccharomyces cerevisiae in the presence of exogenous fatty acid. Journal of Fermentation and Bioengineering.

[10] Madeo F, Frohlich E, Ligr M, Grey M, Sigrist SJ, Wolf DH, Frohlich K-U (1999). Oxygen stress: A regulator of apoptosis in yeast. Journal of Cell Biology.

[11] Ribeiro GF, Corte-Real M, Johansson B (2006). Characterization of DNA damage in yeast apoptosis induced by hydrogen peroxide, acetic acid, and hyperosmotic shock. Molecular Biology of the Cell.

[12] Castro FAV, Mariani D, Panek AD, Eleutherio ECA, Pereira MD (2008). Cytotoxicity mechanism of two naphthoquinones (menadione and plumbagin) in *Saccharomyces cerevisiae*. PLoS One.

[13] Baran I, Ganea C, Scordino A, Musumeci F, Barresi V, Tudisco S, Privitera S, Grasso R, Condorelli D, Ursu I, Baran V, Katona E, Mocanu M-M, Gulino M, Ungureanu R, Surcel M, Ursaciuc C (2010). Effects of menadione, hydrogen peroxide, and quercetin on apoptosis and delayed luminescence of human leukemia Jurkat T-cells. Cell Biochemistry and Biophysics.

[14] Criddle DN, Gillies S, Baumgartner-Wilson HK, Jaffar M, Chinje EC, Passmore S, Chvanov M, Barrow S, Gerasimenko OV, Tepikin AV, Sutton R, Petersen OH (2006). Menadione-induced reactive oxygen species generation via redox cycling promotes apoptosis of murine pancreatic acinar cells. Journal of Biological Chemistry.

[15] Osorio NS, Carvalho A, Almeida AJ, Padilla-Lopez S, Leao C, Laranjinha J, Ludovico P, Pearce DA, Rodrigues F (2007). Nitric oxide signaling is disrupted in the yeast model for Batten disease. Molecular Biology of the Cell.

[16] Herrero E, Ros J, Bellí G, Cabiscol E (2008). Redox control and oxidative stress in yeast cells. Biochimica et Biophysica Acta.

[17] Bilinski T, Lukaszkiewicz J, Sledziewski A (1978). Demonstration of anaerobic catalase synthesis in cz1 mutant of *Saccharomyces cerevisiae*. Biochemical and Biophysical Research Communications.

[18] Koziol S, Zagulski M, Bilinski T, Bartosz G (2005). Antioxidants protect the yeast *Saccharomyces cerevisiae* against hypertonic stress. Free Radical Research.

[19] Millard PJ, Roth BL, Thi HP, Yue ST, Haugland RP (1997). Development of the FUN-1 family of fluorescent probes for vacuole labeling and viability testing of yeasts. Applied and Environmental Microbiology.

[20] Benov L, Sztejnberg L, Fridovich I (1998). Critical evaluation of the use of hydroethidine as a measure of superoxide anion radical. Free Radical Biology and Medicine.

[21] Levine RL, Williams JA, Stadtman EP, Shacter E, Lester P (1994). Carbonyl assays for determination of oxidatively modified proteins. Methods in Enzymology.

[22] Zheng K, Pan J-W, Ye L, Fu Y, Peng H-Z, Wan B-Y, Gu Q, Bian H-W, Han N, Wang J-H, Kang B, Pan J-H, Shao H-H, Wang W-Z, Zhu M-Y (2007). Programmed cell death-involved aluminum toxicity in yeast alleviated by antiapoptotic members with decreased calcium signals. Plant Physiology.

[23] Minois N, Frajnt M, Wilson C, Vaupel JW (2005). Advances in measuring lifespan in the yeast *Saccharomyces cerevisiae*. Proceedings of the National Academy of Sciences of the United States of America.

[24] Carmona-Gutierrez D, Eisenberg T, Buttner S, Meisinger C, Kroemer G, Madeo F (2010). Apoptosis in yeast: Triggers, pathways, subroutines. Cell Death and Differentiation.

[25] Leadsham JE, Kotiadis VN, Tarrant DJ, Gourlay CW (2010). Apoptosis and the yeast actin cytoskeleton. Cell Death and Differentiation.

[26] Esterbauer H, Schaur RJ, Zollner H (1991). Chemistry and biochemistry of 4-hydroxynonenal, malonaldehyde and related aldehydes. Free Radical Biology and Medicine.

[27] Hamann K, Shi R (2009). Acrolein scavenging: A potential novel mechanism of attenuating oxidative stress following spinal cord injury. Journal of Neurochemistry.

[28] Huang Y-J, Jin M-H, Pi R-B, Zhang J-J, Ouyang Y, Chao X-J, Chen M-H, Liu P-Q, Yu J-C, Ramassamy C, Dou J, Chen X-H, Jiang Y-M, Qin J (2013). Acrolein induces Alzheimer’s disease-like pathologies in vitro and in vivo. Toxicology Letters.

[29] Shi R, Rickett T, Sun W (2011). Acrolein-mediated injury in nervous system trauma and diseases. Molecular Nutrition & Food Research.

[30] Ghosh N, Ghosh R, Mandal SC (2011). Antioxidant protection: A promising therapeutic intervention in neurodegenerative disease. Free Radical Research.

[31] D’Alessandro A, Zolla L (2011). The SODyssey: Superoxide dismutases from biochemistry, through proteomics, to oxidative stress, aging and nutraceuticals. Expert Review of Proteomics.

[32] Sturtz LA, Diekert K, Jensen LT, Lill R, Culotta VC (2001). A fraction of yeast Cu, Zn-superoxide dismutase and its metallochaperone, CCS, localize to the intermembrane space of mitochondria: A physiological role for SOD1 in guarding against mitochondrial oxidative damage. Journal of Biological Chemistry.

[33] Lewinska A, Bilinski T, Bartosz G (2004). Limited effectiveness of antioxidants in the protection of yeast defective in antioxidant proteins. Free Radical Research.

[34] Wallace MA, Bailey S, Fukuto JM, Valentine JS, Gralla EB (2005). Induction of phenotypes resembling CuZn-superoxide dismutase deletion in wild-type yeast cells: An in vivo assay for the role of superoxide in the toxicity of redox-cycling compounds. Chemical Research in Toxicology.

[35] Jakubowski W, Biliński T, Bartosz G (1999). Sensitivity of antioxidant-deficient yeast *Saccharomyces cerevisiae* to peroxynitrite and nitric oxide. Biochimica et Biophysica Acta.

[36] Kwolek-Mirek M, Bartosz G, Spickett CM (2011). Sensitivity of antioxidant-deficient yeast to hypochlorite and chlorite. Yeast.

[37] Okazaki S, Tachibana T, Naganuma A, Mano N, Kuge S (2007). Multistep disulfide bond formation in Yap1 is required for sensing and transduction of H_2_O_2_ stress signal. Molecular Cell.

[38] Azevedo D, Tacnet F, Delaunay A, Rodrigues-Pousada C, Toledano MB (2003). Two redox centers within Yap1 for H_2_O_2_ and thiol-reactive chemicals signaling. Free Radical Biology and Medicine.

[39] Zhu Q, Sun Z, Jiang Y, Chen F, Wang M (2011). Acrolein scavengers: Reactivity, mechanism and impact on health. Molecular Nutrition & Food Research.

[40] Adams JD, Klaidman LK (1993). Acrolein-induced oxygen radical formation. Free Radical Biology and Medicine.

[41] Wang X, Mann CJ, Bai Y, Ni L, Weiner H (1998). Molecular cloning, characterization, and potential roles of cytosolic and mitochondrial aldehyde dehydrogenase in ethanol metabolism in *Saccharomyces cerevisiae*. Journal of Bacteriology.

[42] Zarkovic N, Cipak A, Jaganjac M, Borovic S, Zarkovic K (2013). Pathophysiological relevance of aldehydic protein modifications. Journal of Proteomics.

[43] Trotter EW, Collinson EJ, Dawes IW, Grant CM (2006). Old yellow enzymes protect against acrolein toxicity in the yeast *Saccharomyces cerevisiae*. Applied and Environmental Microbiology.

[44] Wonisch W, Kohlwein SD, Schaur J, Tatzber F, Guttenberger H, Zarkovic N, Winkler R, Esterbauer H (1998). Treatment of the budding yeast Saccharomyces cerevisiae with the lipid peroxidation product 4-HNE provokes a temporary cell cycle arrest in G1 phase. Free Radical Biology and Medicine.

[45] Wloch-Salamon DM, Bem AE (2013). Types of cell death and methods of their detection in yeast *Saccharomyces cerevisiae*. Journal of Applied Microbiology.

[46] Eisenberg T, Carmona-Gutierrez D, Büttner S, Tavernarakis N, Madeo F (2010). Necrosis in yeast. Apoptosis.

[47] Giannattasio S, Guaragnella N, Ždralevic M, Marra E (2013). Molecular mechanisms of *Saccharomyces cerevisiae* stress adaptation and programmed cell death in response to acetic acid. Frontiers in Microbiology.

[48] Comporti M (1989). Three models of free radical-induced cell injury. Chemico-Biological Interactions.

[49] Kim I-S, Sohn H-Y, Jin I (2011). Adaptive stress response to menadione-induced oxidative stress in *Saccharomyces cerevisiae* KNU5377. Journal of Microbiology.

[50] Gourlay C, Ayscough K (2005). A role for actin in aging and apoptosis. Biochemical Society Transactions.

[51] Gourlay CW, Ayscough KR (2005). The actin cytoskeleton in ageing and apoptosis. FEMS Yeast Research.

[52] Dalle-Donne I, Carini M, Vistoli G, Gamberoni L, Giustarini D, Colombo R, Maffei Facino R, Rossi R, Milzani A, Aldini G (2007). Actin Cys374 as a nucleophilic target of α, β-unsaturated aldehydes. Free Radical Biology and Medicine.

[53] Gourlay CW, Carpp LN, Timpson P, Winder SJ, Ayscough KR (2004). A role for the actin cytoskeleton in cell death and aging in yeast. Journal of Cell Biology.

[54] Gourlay CW, Ayscough KR (2005). Identification of an upstream regulatory pathway controlling actin-mediated apoptosis in yeast. Journal of Cell Science.

[55] Wang L, Sun Y, Asahi M, Otsu K (2011). Acrolein, an environmental toxin, induces cardiomyocyte apoptosis via elevated intracellular calcium and free radicals. Cell Biochemistry and Biophysics.

[56] Liu F, Li X-L, Lin T, He D-W, Wei G-H, Liu J-H, Li L-S (2012). The cyclophosphamide metabolite, acrolein, induces cytoskeletal changes and oxidative stress in Sertoli cells. Molecular Biology Reports.

[57] Jaganjac M, Tirosh O, Cohen G, Sasson S, Zarkovic N (2013). Reactive aldehydes: second messengers of free radicals in diabetes mellitus. Free Radical Research.

[58] Guéraud F, Atalay M, Bresgen N, Cipak A, Eckl PM, Huc L, Jouanin I, Siems W, Uchida K (2010). Chemistry and biochemistry of lipid peroxidation products. Free Radical Research.

